# P02.110. Effect of traditional acupuncture on circulating endothelial progenitor cells in patients with coronary heart disease

**DOI:** 10.1186/1472-6882-12-S1-P167

**Published:** 2012-06-12

**Authors:** J Painovich, A Phan, N Li, S Chowdhury, S Dhawan, D Taylor, E Marban, C Bairey Merz

**Affiliations:** 1 Cedars Sinai Medical Center, Los Angeles, USA; 2 University of Minnesota, Minneapolis, USA

## Purpose

Endothelial progenitor cells (EPCs) are thought to exert beneficial effects on atherosclerosis, angiogenesis, and vascular repair. We performed a randomized pilot "proof of concept" study of traditional acupuncture (TA) and circulating EPCs in patients with coronary heart disease (CHD).

## Methods

Thirteen subjects were randomized to TA, sham acupuncture (SA) or waiting control (WC) for 12 weeks. TA received treatments at CHD specific acupuncture points while SA received tube pressure with no needle insertion proximate to the TA site but not considered active. WC received no intervention. EPCs at study entry/exit were quantified by flow cytometry using CD34, CD133 and VEGFR2 cell surface markers.

## Results

Eight men and five women (mean age 59±11 yrs) were included. Compared to entry, TA had a significant increase in percentage change from baseline in level of cells expressing CD34+/VEGFR2+ compared to SA and WC (p=0.03). No group differences were evident in immature EPCs expressing CD34+/CD133+, although these were lowest in the TA group; numbers did not correlate with elevation in the CD34+/VEGFR2+group.

## Conclusion

Results suggest that TA may increase mobilization of specific EPC sub-populations (VEGF R2) while simultaneously decreasing levels of a more immature cell type (CD133) and may beneficially alter levels of EPCs. This pilot study provides feasibility and outcome variability data for clinical trial planning purposes. Further studies are warranted to evaluate whether traditional acupuncture can beneficially impact CHD via augmentation of EPC regenerative pathways.

